# Case Report: Modified Taulinoplasty: a new technique for minimally invasive repair of pectus excavatum

**DOI:** 10.3389/fsurg.2023.1343515

**Published:** 2024-01-12

**Authors:** Simone Frediani, Federico Beati, Valerio Pardi, Ivan Pietro Aloi, Arianna Bertocchini, Antonella Accinni, Simone Reali, Paolo Maria Salvatore Schingo, Alessandro Inserra

**Affiliations:** ^1^ General and Thoracic Pediatric Surgery Unit, Bambino Gesù Children’s Hospital, IRCCS, Rome, Italy; ^2^ Department of Anesthesia and Critical Care, Bambino Gesù Children’s Hospital, IRCCS, Rome, Italy; ^3^ Department of Imaging, Bambino Gesù Children’s Hospital, IRCCS, Rome, Italy

**Keywords:** Taulinoplasty, pectus up, pectus excavatum, thoracic wall malformation, funnel chest

## Abstract

**Introduction:**

About 95% of congenital chest wall deformities are pectus abnormalities, with pectus excavatum (PE) being the most common. The purpose of this work is to offer a modified Taulinoplasty Technique based on 35 consecutive PE patients' 1-year single-center experience in 2022.

**Technique:**

One minimally invasive procedure for PE is taulinoplasty. In order to prevent invasion of the mediastinum or pleural cavity, it is considered that external traction can be used to raise the sternum. Our experience indicates that the most common surgical consequences of this procedure—which involves creating a submuscular and subcutaneous tunnel to install the metal device—are post-operative seroma and wound dehiscence. We modified the conventional method to achieve more aesthetically pleasing results.

**Discussion:**

Taulinoplasty seems to be a safe technique, easier and more feasible than standard Taulinoplasty, with better outcomes in terms of surgical complications, although further experience is necessary to confirm our preliminary data.

## Introduction

Pectus abnormalities account for about 95% of congenital chest wall defects, with pectus excavatum (PE) being the most frequent. A “funnel chest” or depression of the anterior chest wall is a hallmark of pectus excavatum. Although the third to seventh costo-cartilages, or ribs, are affected, the xiphisternum is where the deformity is most severe. PE presents in most cases as an asymmetrical defect, even though symmetrical deformities are described. An infant's pectus deformity may be noticed at birth or emerge later in infancy (1). The aim of this work is to present a modified Taulinoplasty Technique (2) based on a 1-year single-centre experience of 35 patients consecutively treated for PE in 2022.

## Technique

Taulinoplasty is a minimally invasive technique for PE. It is assumed that the lifting of the sternum can be accomplished through external traction in order to avoid invading the mediastinum or pleural cavity (https://youtu.be/u-a-vEd3hBM) (2). According to our experience, post-operative seroma and wound dehiscence are the most frequent surgical complications of this procedure, in which a submuscular and subcutaneous tunnel are created in order to place the metal device.

We modified the standard technique as follows to improve aesthetic results and outcomes.

Informed consent was achieved from patients/parents before procedure (standard or modified).

**Figure 1:**
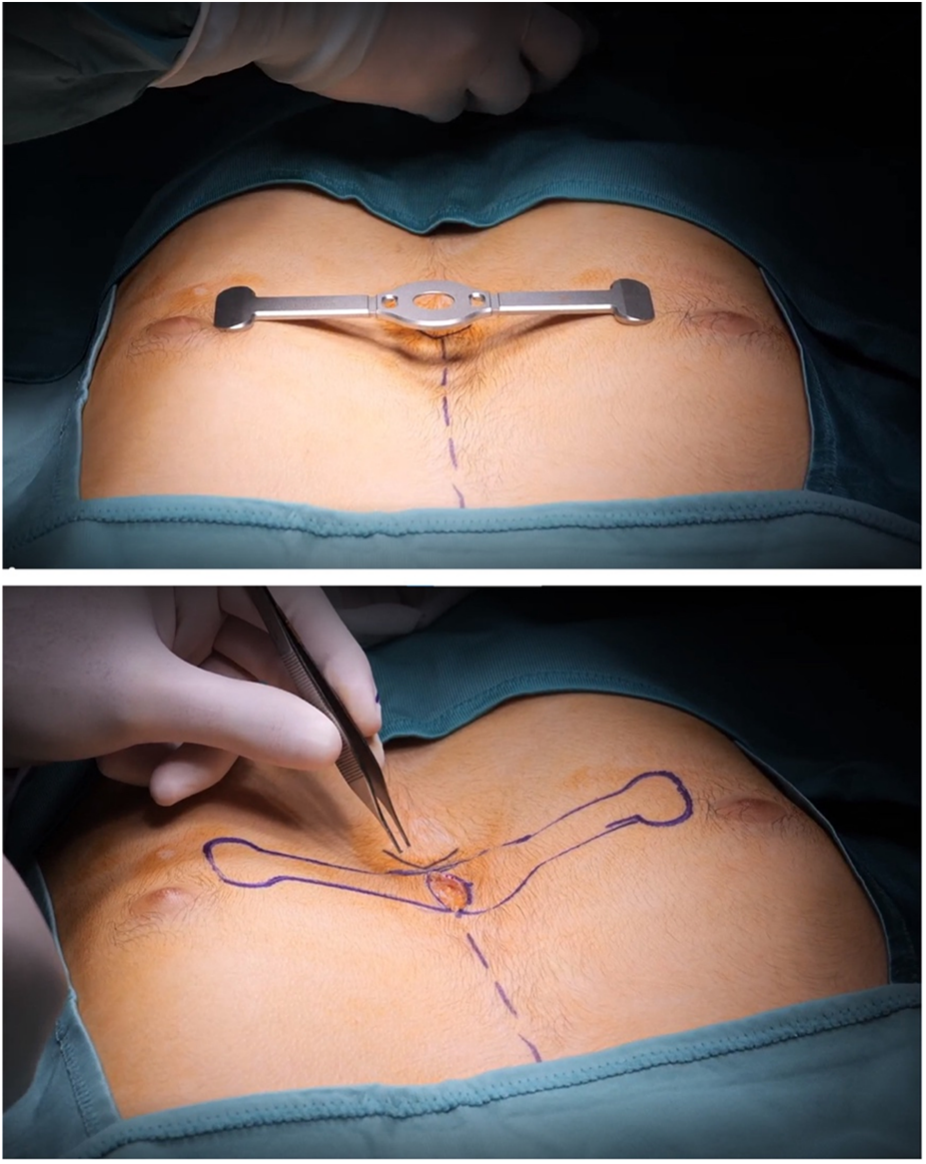
Vertical incision.

**Figure 2:**
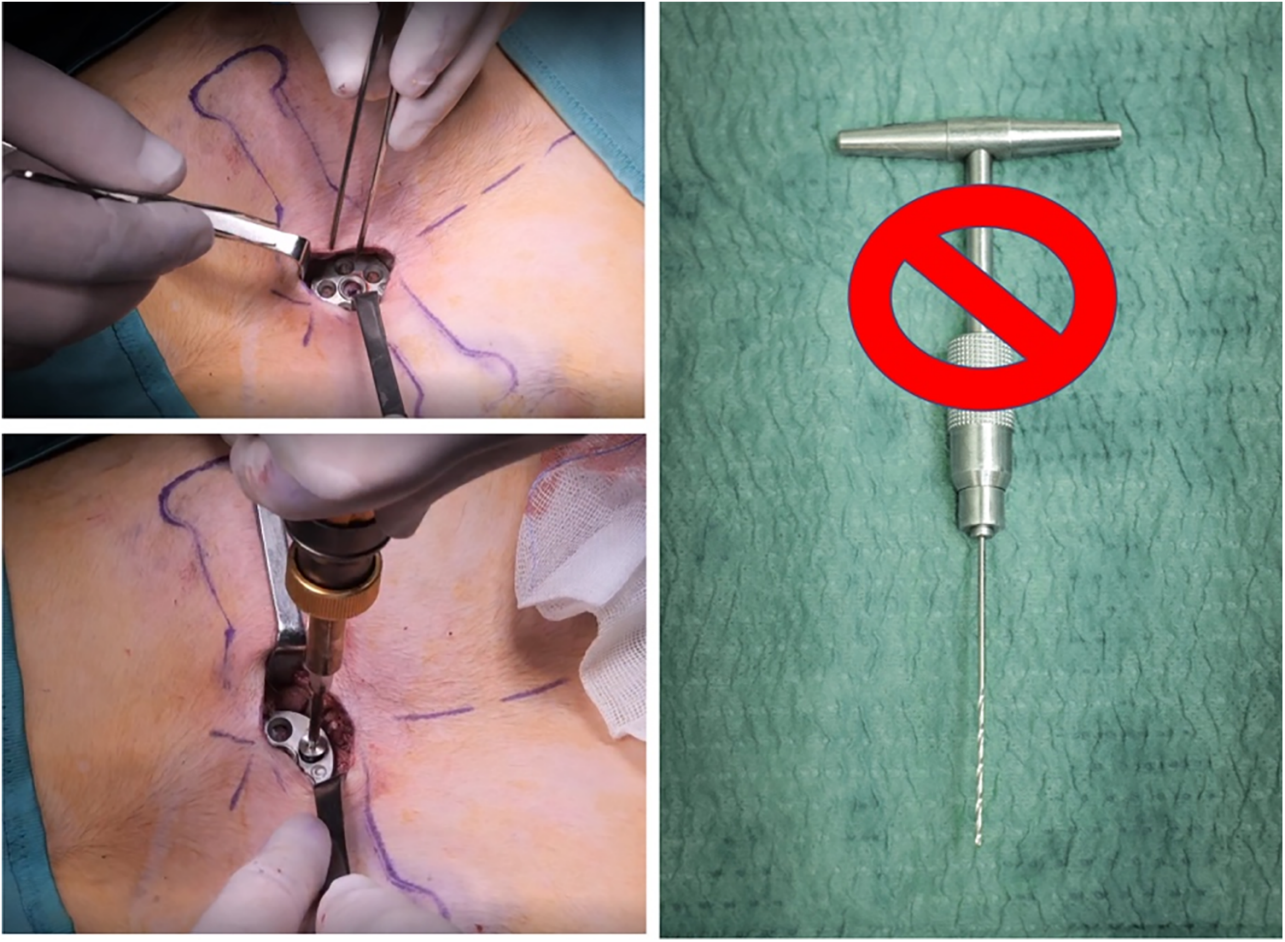
Self-tapping screws on the sternal plate without surgical drill.

**Figure 3:**
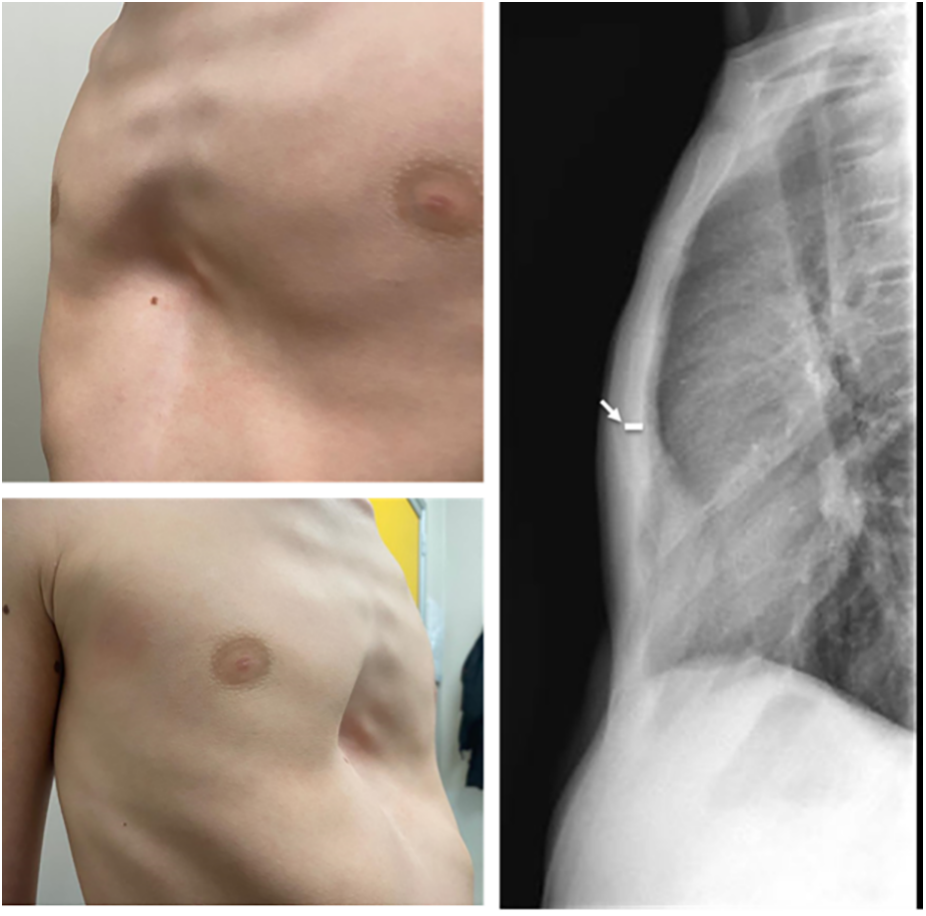
X-ray chest to evaluate the thickness of the sternum.

1) Standard technique provides a transverse parasternal incision at the major sternal defect in order to introduce the metal device. In our experience, among those who underwent standard surgical procedures, 5/11 (45%) patients had a large post-operative seroma. Based on seroma size we evaluated the need of needle drainage of it; no drainage was left in place. One patient had wound dehiscence after seroma. Therefore, we placed a Sylastic® sheet under the surgical wound, but unfortunately, this didn't modify the incidence of seroma and increased the risk of wound infection, forcing us to remove it.
2) According to our centre's thoracic surgery experiences, we modified skin incisions from horizontal to vertical, which seems to reduce the risk of post-operative seroma and wound dehiscence. We believe that horizontal incision led to more tension on the wound because of different suture direction of the underlying muscular plane. Vertical incision allows a more anatomical closure of surgical planes, reducing “dead space” between muscle and skin (Figure 1). We did not have post-operative seroma nor keloids of the sternal wound, which according to the international literature account for 10%–20%.
3) Standard Taulinoplasty provides the placement of two-metal back-up stitches laterally to the device in order to reinforce the anchorage to the sternum; we reserved this step only in the case of a severe asymmetrical sternal defect, replacing the metal stitches with synthetic nonabsorbable braided stitches. No device displacements were recorded.
4) We eliminated the use of the surgical drill used to insert the self-tapping screws on the sternal plate, because they are characterized by a drill tip free of threads and configured to cut bone. This modification reduced the risk of bleeding and post-operative seroma (Figure 2). If the screw does not have enough grip, it is possible to angle it slightly so as to have greater resistance.
5) In cases where the xiphoid process is particularly arched, we place a synthetic, nonabsorbable braided suture to anchor it to the bar. This allows for a more natural and aesthetic correction of the defect.
6) The standard technique involves the use of a preoperative chest CT scan to evaluate the thickness of the sternum and, consequently, providing proportional anchoring screws. In the era of patient protection from radiation, we replaced the CT scan with a chest x-ray performed in lateral-lateral projection at a fixed distance of 1.5 m. The patient's arms are extended backwards so as not to interfere with the view of the sternum (1) (Figure 3).

## Discussion

Multiple repairs have been developed for the correction of pectus excavatum, but none has been uniformly accepted as the optimal procedure; this would suggest that none of the alternatives provide perfect results. The current standard of repair is frequently attributed to the Nuss technique or MIRPE (minimally invasive repair of pectus excavatum). This procedure involves inserting a retrosternal metal rod thoracoscopically using two small lateral thoracic incisions (3).

Before MIRPE, the most commonly used technique was the one according to Ravitch, described for the first time in 1949. The procedure includes excision of all deformed costal cartilages with the perichondrium, division of the xiphoid from the sternum, division of the intercostal bundles from the sternum, and transverse sternal osteotomy displacing the sternum anteriorly. His technique was later modified to preserve the perichondrial sheath (4).

**Figure 4:**
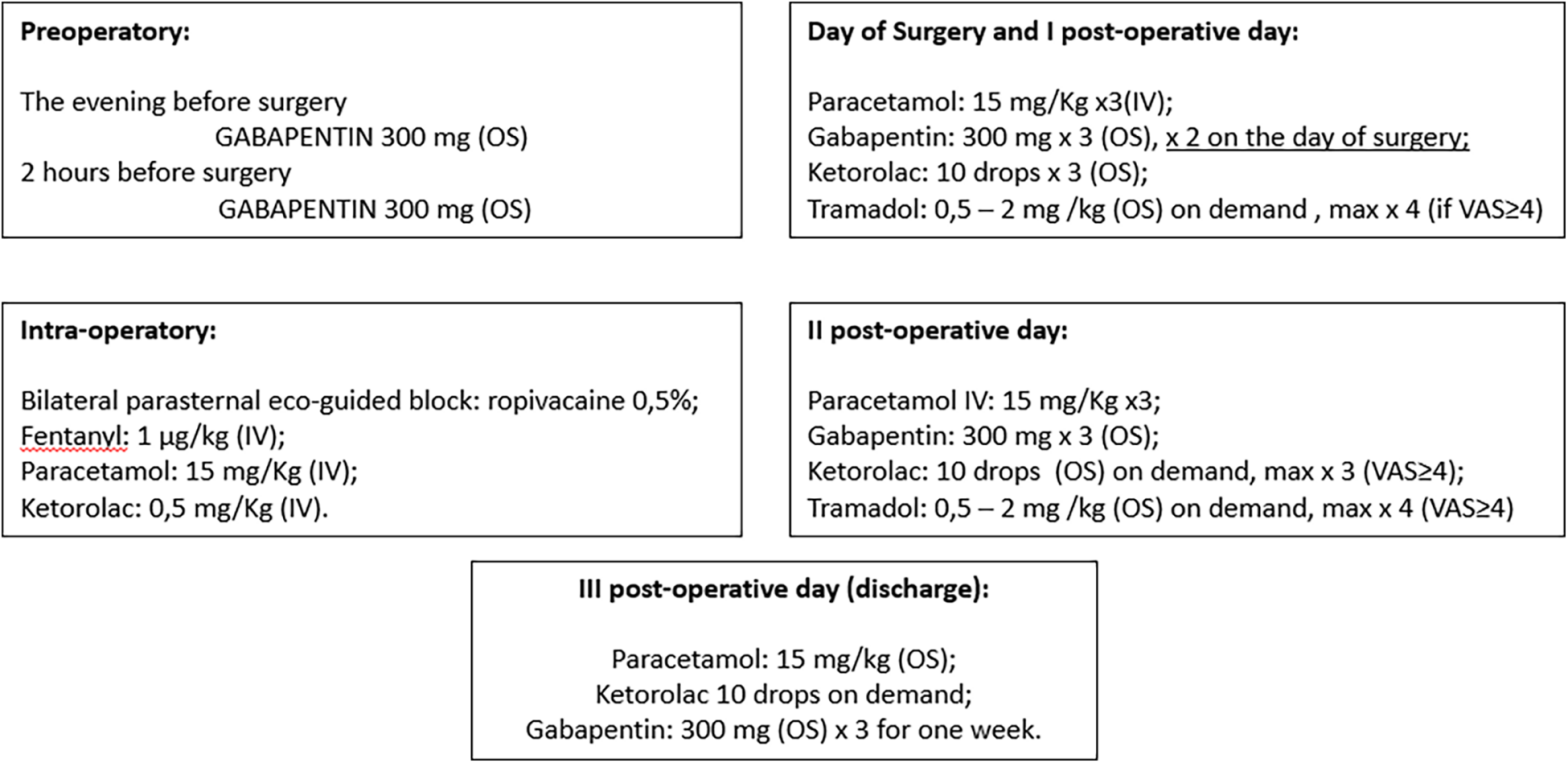
Pain management protocol for taulinoplasty (OS, oral somministration; VAS, visual analogue scale for pain).

A recent paper demonstrates Taulinoplasty as a safe and effective extrathoracic surgical procedure for PE. By avoiding the invasion of the mediastinum and the pleural cavity, it reduces the risk of injury to the intrathoracic vital organs. It also showed significant reductions in hospital length of stay, surgical time, and IV analgesia compared with the MIRPE technique (5, 6). We report our protocol for pain management compared to MIRPE (Figure 4). In this single-centre pilot study, modified Taulinoplasty seems to be a safe technique, easily reproducible with a fast-learning curve and more feasible than standard Taulinoplasty. We report better outcomes than standard technique in terms of surgical complications, although further experience is necessary to confirm our preliminary data.

## Data Availability

The original contributions presented in the study are included in the article/Supplementary Material, further inquiries can be directed to the corresponding author.
